# Genetically Prioritized Plasma Proteins as Candidate Therapeutic Targets for Dry Age-Related Macular Degeneration

**DOI:** 10.1155/joph/3972293

**Published:** 2025-09-30

**Authors:** Jiabao Hou, Ju Guo, Wenlong Shen, Dejian Xie, Ping Li, Yan Zhang, Zhihu Zhao

**Affiliations:** ^1^Laboratory of Advanced Biotechnology, Beijing Institute of Biotechnology, Beijing, China; ^2^Department of Ophthalmology, Tianjin Medical University General Hospital, Tianjin, China

**Keywords:** drug development, dry age-related macular degeneration, Mendelian randomization, plasma proteins, scRNA-seq

## Abstract

**Background:**

Dry age-related macular degeneration (AMD) is a leading cause of vision loss in the elderly, yet effective treatments remain limited. This study aimed to identify putative causal plasma proteins linked to dry AMD through proteome-wide Mendelian randomization (MR) and genetic pleiotropy analyses.

**Methods:**

We performed proteome-wide MR analyses using protein quantitative trait loci (pQTL) data from the UK Biobank Pharma Proteomics Project (UKB-PPP) and genetic summary statistics for dry AMD from FinnGen R11. Replication analyses were conducted using pQTL data from the deCODE Genetics cohort and dry AMD GWAS data from the Million Veteran Program (MVP), all in individuals of European ancestry. To enhance robustness, we conducted additional sensitivity analyses using colocalization and summary data–based MR (SMR) approaches. Cell-type-specific expression profiles derived from single-cell RNA sequencing (scRNA-seq) data were used to prioritize candidate drug targets based on tissue relevance and druggability.

**Results:**

Discovery MR analysis identified 22 plasma proteins putatively associated with dry AMD. Replication MR tests and genetic pleiotropy analyses prioritized 12 proteins. Two retinal cell–specific genes were validated through scRNA-seq analysis. Druggability assessment confirmed C3 as an established AMD target and identified MASP1 and CFHR2 as complement pathway components with partial druggability. Notably, the remaining nine proteins represent novel pathways in dry AMD pathogenesis, four of which offer immediate drug-repurposing opportunities with approved agents, while five represent previously unexplored therapeutic candidates with high mechanistic plausibility.

**Conclusions:**

This study provides genetically supported therapeutic candidates for dry AMD and prioritizes candidates with high clinical potential, advancing therapeutic strategies for dry AMD.

## 1. Introduction

Age-related macular degeneration (AMD) is one of the leading causes of irreversible blindness in older adults, accounting for approximately 8.7% of global visual impairment and projected to affect nearly 288 million people by 2040 [1, 2]. Dry AMD, also known as non-neovascular or atrophic AMD, accounts for 85%–90% of all AMD cases and is characterized by progressive degeneration of the retinal pigment epithelium and photoreceptors [3]. Aging is the predominant risk factor for the development of dry AMD [4]. Consequently, with the increase in life expectancy, the progressive and irreversible nature of dry AMD has led to a substantial societal economic burden and an increased consumption of healthcare resources in the aging population [5–7]. To date, the U.S. Food and Drug Administration (FDA) has approved of Syfovre (pegcetacoplan, targeting complement C3) in February 2023 and Iverzay (avacincaptad pegol, targeting complement C5) in August 2023 for clinical use in patients with dry AMD which are far from meeting the extensive clinical needs [8, 9]. Furthermore, because the complement system plays a pivotal role in the innate immune response, prolonged complement suppression can increase the vulnerability of patients to specific pyogenic infections or lead to adverse events [10]. Therefore, there is a pressing need to accelerate the development and research of new therapeutic targets and drugs that are less likely to disrupt the balance of the immune system.

Plasma proteins are crucial components in a wide array of biological processes (BPs) and represent a significant reservoir of potential therapeutic targets for medical interventions [11, 12]. Statistically, 75% of FDA-approved drugs target human proteins. In the context of AMD, recent advances in proteomic technologies have enabled the identification of promising protein biomarkers and potential pathogenic pathways. Guo et al. conducted a meta-analysis focusing on mass spectrometry–based proteomic profiling of ocular fluids, including aqueous and vitreous humor, across 11 studies. Their findings highlighted serotransferrin (TF), apolipoprotein A1 (APOA1), complement C3 (C3), and lipocalin-1 (LCN1) as candidate biomarkers significantly associated with AMD pathogenesis [13]. In a broader investigation, Sideri et al. systematically reviewed 22 unbiased proteomic studies using both mass spectrometry and aptamer-based approaches across various tissues and biofluids in dry and neovascular AMD. Their analysis, encompassing 6932 participants, revealed recurrent alterations in lipid metabolism, complement activation, protease regulation, and extracellular matrix (ECM) remodeling [14]. However, the current landscape of AMD proteomics is limited by low sample size and statistical bias. Therefore, there is an urgent need for larger-scale, statistically rigorous investigations to advance our understanding of AMD proteomics and to identify reliable biomarkers for early detection and intervention.

A recent genome-wide association study (GWAS) of plasma proteomics provided a detailed mapping of quantitative protein trait loci (pQTL) for 2923 proteins, identifying 14,287 primary genetic associations [15]. These genetic associations offer a valuable opportunity to systematically assess the putative causal relationship between potential drug targets and the human disease phenotypes using genetic approaches, such as Mendelian randomization (MR) [16]. MR uses genetic variants as instrumental variables (IVs) to mimic observational data of randomized controlled trials, enabling researchers to assess causal relationships between exposures and outcomes in a more controlled manner, independent of environmental confounding factors and reverse causality bias [17–19]. This approach integrates genetic information from existing large cohorts, avoids redundant work, significantly shortens the drug development cycle, and accelerates the development and clinical application of new drugs [20, 21]. Recent advancements in proteomics and genomics have significantly enhanced our understanding of the genetic mechanisms of complex diseases. For instance, Emilsson et al. demonstrated that numerous serum proteins carrying genetic markers coincide with those implicated in a variety of diseases. Their research identified 2021 independent exome array variants associated with serum levels of 1942 proteins, underscoring the potential of serum proteins as both biomarkers and causal agents across a broad spectrum of diseases. This research is crucial for advancing our understanding of the proteogenomic landscape of AMD [16]. In the context of AMD, Han et al. conducted metabolite genome-wide association studies (mGWAS) utilizing multiethnic genetic and metabolomic data from up to 28,000 participants. Their findings highlight the significant contribution of plasma metabolites, particularly those involved in lipid-related pathways and genes, to AMD risk. Moreover, they identified numerous potential causal links between metabolites and AMD, offering valuable insights into the disease's underlying mechanisms [22]. Similarly, Jingzhi et al. integrated MR with cross-sectional analysis from the National Health and Nutrition Examination Survey (NHANES) to investigate 35 biomarkers. Their findings revealed that triglycerides (TG) had a protective effect, while high-density lipoprotein cholesterol (HDL-C) and C-reactive protein (CRP) increased AMD risk, with lipid-related biomarkers more strongly associated with early AMD and CRP with late AMD [23]. Likewise, Zhang et al. employed MR techniques to identify 21 blood proteins potentially causally associated with diabetic kidney disease (DKD). External validation of their findings supported the role of four proteins as promising drug targets, demonstrating the utility of proteomic MR approaches in identifying novel therapeutic targets [21].

Building upon these foundational studies, we performed a comprehensive proteome-wide MR and genetic pleiotropy analyses to explore potential causal links between plasma proteins and dry AMD. The study had three primary objectives: (i) to leverage plasma protein data from two large cohorts to systematically assess the potential causal relationships between circulating proteins and dry AMD risk within a two-sample MR framework, thereby generating hypotheses about underlying biological mechanisms; (ii) to identify shared genetic variants that are associated with both protein levels and dry AMD, providing supporting evidence for potential mechanistic links; and (iii) to connect these genetically supported protein–disease associations with existing or potential therapeutic targets, facilitating future drug discovery and repurposing efforts.

## 2. Methods and Materials

### 2.1. Proteomic Data Source

At the discovery stage, our study leveraged a robust dataset from UKB-PPP, encompassing the proteomic profiles of 2923 plasma proteins from 54,219 participants [15]. To further replicate our analysis, we incorporated additional proteomic data from a study published in Nature Genetics by the deCODE Genetics team, which included data for 4907 plasma protein and 18,084 associations between sequence variants and plasma protein levels of 35,559 Icelanders [24]. To ensure elucidation of a more immediate and precise biological impact on proteins, we exclusively used cis-pQTLs as IVs for MR analysis [25]. This approach was supported by empirical evidence from UKB-PPP data showing that cis-pQTLs exhibited stronger average effects on protein levels (explaining 20.5% of total heritability through 1955 associations, ∼0.0105% per association), compared to trans-pQTLs (10.4% heritability through 12,332 associations, ∼0.00084% per association) [15]. Specifically, we selected cis-pQTLs located within a 1-Mb range of genes that encode the proteins of interest, with a significance threshold set at *p* < 5E − 8.

### 2.2. Outcome Data Sources

In the discovery phase, summary data from GWAS on dry AMD for this study were obtained from the FinnGen Release 11, which included data for 306,075 participants (7589 cases and 272,504 controls). This dataset can be accessed from https://www.finngen.fi/en. For the replication phase, summary statistics of dry AMD were obtained from the Million Veteran Program (MVP) European-ancestry cohort (27,144 cases and 412,580 controls). These data were accessed through the database of Genotypes and Phenotypes (dbGaP) under accession number phs002453.v1.p1. The complete dataset is publicly available at https://ftp.ncbi.nlm.nih.gov/dbgap/studies/phs002453/phs002453.v1.p1/analyses/GIA/MVP_R4.1000G_AGR.GIA.PheCodes_SenseOrgans_batch1/MVP_R4.1000G_AGR.Phe_362_21.EUR.GIA.dbGaP.txt.gz.

### 2.3. MR Analysis

Guided by the principles set forth in Strengthening the Reporting of Observational Studies in Epidemiology Mendelian Randomization (STROBE-MR), we used the R package “TwoSampleMR” (v 0.5.6) to investigate the association between plasma proteins and dry AMD in our study [26, 27]. To identify qualified genetic instruments, we extracted variants that met the three core IV assumptions under stringent criteria: (1) relevance: genetic variants that reached genome-wide significance (*p* < 5E − 8) were selected as IVs, (2) independence: a minimum physical distance of 1000 kb and LD of *r*^2^ < 0.1, using the UK10K reference panel [28], (3) exclusion restriction: to evaluate potential violations of this assumption, we assessed heterogeneity using Cochran's Q test [29] and evaluated horizontal pleiotropy using the MR-Egger intercept [30]. The presence of heterogeneity or pleiotropy, as indicated by *p* values < 0.05 in these tests, informed our selection of analytical methods. For proteins with a single instrumental SNP, we applied the Wald ratio method [31]. In the absence of pleiotropy or heterogeneity, causal estimates were derived using the inverse variance weighted (IVW) method [32]. In the presence of heterogeneity, we prioritized the method yielding the largest *p* value among the weighted median and multiplicative random-effects IVW (mre-IVW) approaches [33, 34]. When pleiotropy was detected, the primary estimate was based on the method with the highest *p* value between MR-Egger [35] and MR-PRESSO (Pleiotropy RESidual Sum and Outlier). If both heterogeneity and pleiotropy were present, we reported the estimate corresponding to the largest *p* value among the weighted median, mre-IVW, MR-Egger, and MR-PRESSO methods. Multiple testing correction was performed using the Bonferroni method, with statistical significance set at *p* < 0.05 divided by the number of tests. This study was conducted and reported in accordance with the STROBE-MR guideline. The completed STROBE-MR checklist is available in Supporting data 1.

### 2.4. Sensitivity Analysis

As the first component of our sensitivity analyses, we replicated the main findings from the discovery MR analysis (UKB-PPP proteins and FinnGen R11 dry AMD) in three additional cohort combinations: deCODE proteins with FinnGen R11 dry AMD, deCODE proteins with MVP dry AMD, and UKB-PPP proteins with MVP dry AMD. Additionally, we calculated the *F*-statistic to detect bias arising from weak IVs, considering an *F*-statistic < 10 as indicative of weak instruments [36]. The *F*-statistic for each IV was calculated as

(1) $$\begin{array}{c} F=\frac{R^{2}\times \left(N-K-1\right)}{\left(1+R^{2}\right)\times K}, \end{array}$$

where *R*^2^ = 2 × EAF × (1 − EAF) × *β*^2^, EAF indicates effect allele frequency; β: SNP-exposure effect, *N* is sample size, and *K* is instrument count. We excluded all proteins lacking instruments with *F* > 10.

To further assess the robustness of the primary MR results, we employed complementary mode-based estimators [37], including the weighted mode and simple mode methods. We additionally applied a Bayesian colocalization approach to evaluate whether the drug target proteins and dry AMD shared a common causal variant within specific genomic regions. This analysis was performed using the R package coloc (v3.1), with a posterior probability (PP4) > 0.80 considered as evidence of a shared causal variant [38]. To further support the potential causal relationship between pQTLs and dry AMD, we conducted summary-based MR (SMR) analysis [39], accompanied by the HEIDI test. The HEIDI test leverages multiple SNPs to distinguish pleiotropic effects from those due to linkage disequilibrium (LD). Analyses were conducted using SMR software (v1.3.1), with statistical significance set at a *p* < 0.05 divided by the number of tests for SMR. A HEIDI *p* value > 0.05 indicated that the observed protein–dry AMD associations were unlikely to be driven by LD. The study protocol was not preregistered.

### 2.5. Assessment of Horizontal Pleiotropy

To evaluate horizontal pleiotropy in our MR analyses, we employed multiple complementary approaches. First, we examined the MR-Egger regression intercept, which tests for directional pleiotropy by estimating whether the genetic instruments have a nonzero average pleiotropic effect [40]. Second, we implemented the MR-PRESSO framework to detect and correct for outlier variants that may bias causal estimates due to horizontal pleiotropy [41]. When pleiotropy was detected (based on either the MR-Egger intercept or the MR-PRESSO global test), we reported the causal estimate from the method with the highest *p* value between MR-Egger test and MR-PRESSO outlier-corrected test, prioritizing more robust results. In addition, for proteins showing significant colocalization or SMR signals with dry AMD, but also exhibiting strong evidence of pleiotropy, we cautiously interpret these associations as likely driven by pleiotropic effects rather than putative causal associations.

### 2.6. Pathway and Functional Enrichment Analysis

We next conducted a comprehensive enrichment analysis using the Gene Ontology (GO) and the Kyoto Encyclopedia of Genes and Genomes (KEGG) through the KEGG Orthology-Based Annotation System (KOBAS) to elucidate the biological roles and metabolic pathways associated with our cohort of differentially expressed proteins [42, 43]. GO analysis, a robust method to examine the collective attributes of genes across BPs, molecular functions (MF), and cellular components (CC), was used to identify significant enrichment within the GO annotations [44, 45]. Our KEGG analysis focused on the metabolic pathways to which these genes contribute. We performed this enrichment analysis on significant proteins in discovery MR tests, retaining only those results with a corrected *p* value of less than 0.05 to ensure statistical relevance.

### 2.7. PPI Network and the Identification of Druggable Proteins

PPI network was conducted by utilizing the STRING database, followed by the generation of a subnetwork diagram utilizing Cytoscape for visual representation and analysis [46, 47]. To pinpoint these central gene clusters, we employed the Cytoscape software, utilizing its cytoHubba and MCODE plugins for a robust analysis. Subsequently, we evaluated their potential as therapeutic targets by examining their interactions with drugs using DGIdb, ChEMBL, and DrugBank [48–50]. This process prioritizes the druggable targets by consolidating data on drug–gene interactions, gene functionality, text mining, and expert knowledge curation and documents the relevant drug names and stages of drug development targeting the identified proteins.

### 2.8. Single Cell-Type Expression Analysis

To further assess the specific expression of candidate drug targets by cell type, we analyzed single-cell RNA sequencing (scRNA-seq) data from the retinal tissues of 6 healthy controls (GSM6841143-GSM6841148) and 4 dry AMD patients (GSM6841156-GSM6841159), downloaded from the GSE221042 array [51]. We utilized the R package “Seurat” (v5) along with “harmony” to integrate and analyze the scRNA-seq dataset [52, 53]. Initial quality control removed genes detected in fewer than three cells and cells meeting any of the following criteria: fewer than 200 detected genes, greater than 7500 detected genes, or mitochondrial gene fraction (mt%) > 10%. We applied SCTransform (SCT) for normalization and variance stabilization, regressing out sequencing depth and mitochondrial percentage where appropriate. To detect and remove doublets, we applied DoubletFinder and removed predicted doublets prior to downstream analyses. For integration and batch correction across samples, we used Harmony on the SCT-corrected PCA embeddings. Clustering was performed on the Harmony-corrected principal components using the Louvain algorithm, with cluster resolution selected to balance granularity and interpretability. For cell type annotation, we referred to the cell clusters and marker genes from the original literature [51]. Differential expression analysis was conducted using the Wilcoxon rank sum test to compare gene expression levels between individual cell types and a composite of all other cell types. Genes were considered enriched in a particular cell type if they exhibited an average Log2 fold change (Log2FC) > 0.5 and an FDR-adjusted *p* value < 0.05. Additionally, we cross-referenced our findings with differential gene expression data from a study involving 43 geographically atrophy induced pluripotent stem cell (iPSC)–derived retinal pigment epithelium (RPE) cell lines and 36 control iPSC-derived RPE cell lines [54].

## 3. Results

### 3.1. Proteome-Wide MR Analysis Reveals 22 Plasma Proteins Causally Associated With Dry AMD

**Figure 1:**
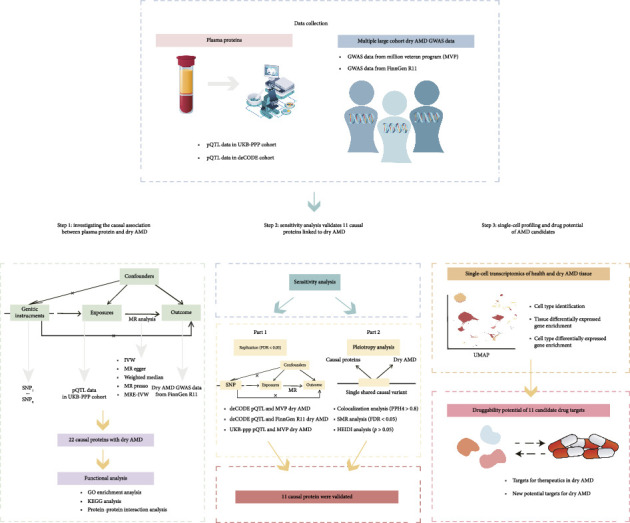
Schematic overview of the study design. Plasma protein data were collected from the UK Biobank Pharma Proteomics Project (UKB-PPP) and deCODE Genetics, along with dry AMD GWAS data from the Million Veteran Program (MVP) and FinnGen Release 11 (R11). In the Discovery stage, a proteome-wide MR analysis was performed using UKB-PPP and FinnGen R11 data to identify causal relationships between plasma proteins and dry AMD. Subsequently, functional analyses, including enrichment analysis and protein–protein interaction analysis, were conducted based on the identified causal proteins. The findings were then validated using various pQTL and GWAS datasets in the replication stage. Colocalization analyses, alongside SMR and HEIDI, were conducted to evaluate confounders and shared causal variants. Potential causal proteins that passed through the validation or colocalization analyses were recognized as candidate drug targets. These candidate drug targets underwent further assessment, including their expression in single-cell RNA-seq data from GSE221042, as well as an evaluation of their druggability and potential as drug targets. Ethical approval for the proteomic, genetic and scRNA-seq data were obtained from the respective institutional review boards, and no additional ethical approval was necessary for this study.

**Figure 2:**
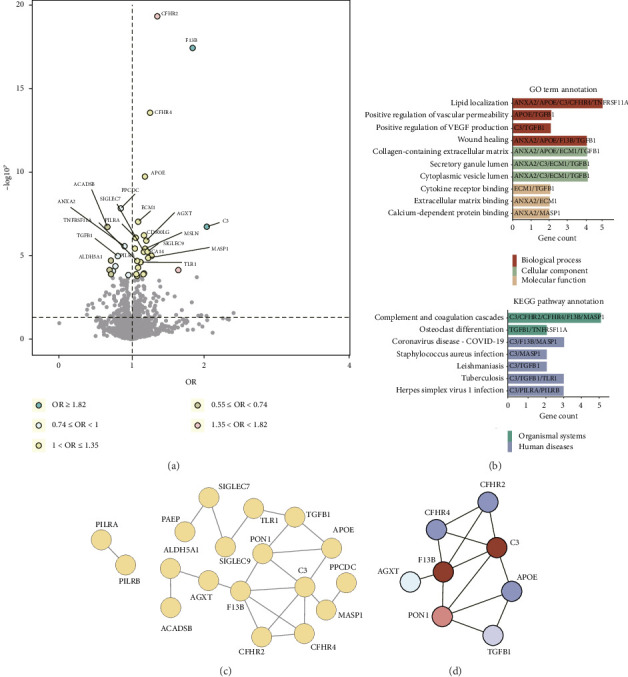
Twenty-two putative causal proteins with dry AMD identified by proteome-wide MR. (a) Volcano plot showed that 22 plasma proteins were associated with the dry AMD risk in the discovery cohort, with varying colors indicating degree of positive and negative correlations and the number of implicated genes. (b) Bar graphs unveiled the top significant GO enrichment and KEGG pathway annotation terms based on 22 identified causal proteins. (c) The protein-protein interaction (PPI) network illustrated a visual representation of the intricate connections among 22 identified causal proteins. Each node represents an individual protein, with connecting lines signifying the interactions between them. (d) The PPI network diagram delineated the extensive interactions among core proteins significantly involved in diverse interactions across four pivotal pathways. Utilizing Cytoscape software, with the cytoHubba and MCODE plugins, we pinpointed clusters of central genes.

An overview of the study design is shown in Figure 1. To uncover potential therapeutic targets for dry AMD, we tested the causal relationships between 2923 plasma proteins and dry AMD by leveraging summary statistics from nonoverlapping sources: the exposure data from the UKB-PPP and the outcome data from the FinnGEN R11 GWAS, thereby minimizing biases such as winner's curse. After stringent quality control and the selection of qualified IVs, valid MR estimates were obtained for 1763 proteins, which we used as the denominator for Bonferroni correction. Our MR analysis identified 22 plasma proteins causally linked to dry AMD (*p* < 0.05/1763), with C3 showing the most significant positive effect (odds ratio [OR] = 2.04 [1.56, 2.66], *p* = 1.87E − 07) (Figure 2(a)). Elevated levels of AGXT, APOE, and F13B were associated with increased risk, while lower levels of ACADSB, ANXA2, TGFB1, and PPCDC were linked to higher risk (Supporting Tables 1-2).

Functional analysis revealed significant enrichment in BPs such as positive regulation of vascular permeability. The collagen-containing ECM, crucial for macular support, was enriched in CC terms. KEGG pathway analysis highlighted involvement in complement and coagulation cascades, suggesting therapeutic targets in dry AMD (Figure 2(b)). PPI network analysis showed that TGFB1 is linked to the collagen-containing ECM pathway, potentially driving AMD-associated fibrosis. C3 was critical within the complement system. APOE and TGFB1 were pivotal in positive regulation of vascular permeability (Figure 2(c)). Additionally, 8 hub genes exhibited extensive interactions with the 22 putative causal proteins, indicating central roles in the gene network (Figure 2(d)).

### 3.2. Sensitivity Analysis Supports the Causality of 12 Proteins With Dry AMD

To validate the 22 putative causal proteins identified in the discovery cohort, we performed multiple sensitivity analyses to assess the robustness of the associations between genetically predicted plasma protein levels and the risk of dry AMD. Specifically, we conducted replication MR analyses using three independent dataset combinations (Figure 1). These analyses consistently validated the associations for 12 proteins, reaching statistical significance after Bonferroni correction (Supporting Tables 3–5). The genetically predicted ORs per standard deviation (SD) increase in protein levels were as follows (Table 1 and Supporting Table 6): C3 at 2.04 (95% CI 1.56–2.66), CFHR2 at 1.36 (95% CI 1.27–1.46), F13B at 1.84 (95% CI 1.61–2.10), MASP1 at 1.23 (95% CI 1.12–1.36), SIGLEC7 at 1.17 (95% CI 1.09–1.27), TLR1 at 1.13 (95% CI 1.06–1.22), PILRB at 1.05 (95% CI 1.03–1.08), PILRA at 1.06 (95% CI 1.03–1.08), APOE at 1.19 (95% CI 1.13–1.26), and ECM1 at 1.09 (95% CI 1.06–1.13). In contrast, we observed a protective association for ANXA2 at 0.90 (95% CI 0.87–0.94) and TGFB1 at 0.81 (95% CI 0.73–0.90). All reported associations derive exclusively from instruments exceeding the *F* > 10 threshold. Of 2923 initially tested proteins, 1763 (60.3%) contained ≥ 1 qualified instrument (*F* > 10) and were retained for discovery analyses (Supporting Table 7). Replication analyses included 13–18 proteins meeting this criterion (Supporting Table 7). MR-PRESSO analysis (Supporting Table 8) supported the absence of significant horizontal pleiotropy for most associations. Additionally, C3 and TLR1 showed strong evidence of genetic co-localization with dry AMD risk (posterior probability for shared causal variant, PPH4 > 80%), suggesting a high likelihood of shared causal variants between protein expression and dry AMD (Table 1 and Supporting Table 9). To further verify the observed findings, we performed SMR and HEIDI tests on 22 proteins. TGFB1, TLR1, and SIGLEC7 passed both SMR (*P* < Bonferroni-corrected threshold) and HEIDI (*p* > 0.05) tests, indicating strong evidence of causality without confounding from LD (Table 1 and Supporting Table 10). Collectively, these comprehensive sensitivity analyses robustly support the potential causal association of 12 plasma proteins with dry AMD risk. To ensure the rigor and transparency of our methodological reporting, the completed STROBE-MR checklist has been included in Supporting Table 11.

### 3.3. Cell-Specific Expression Profile of 12 Causal Genes in Retinal Tissue of AMD Patients

**Figure 3:**
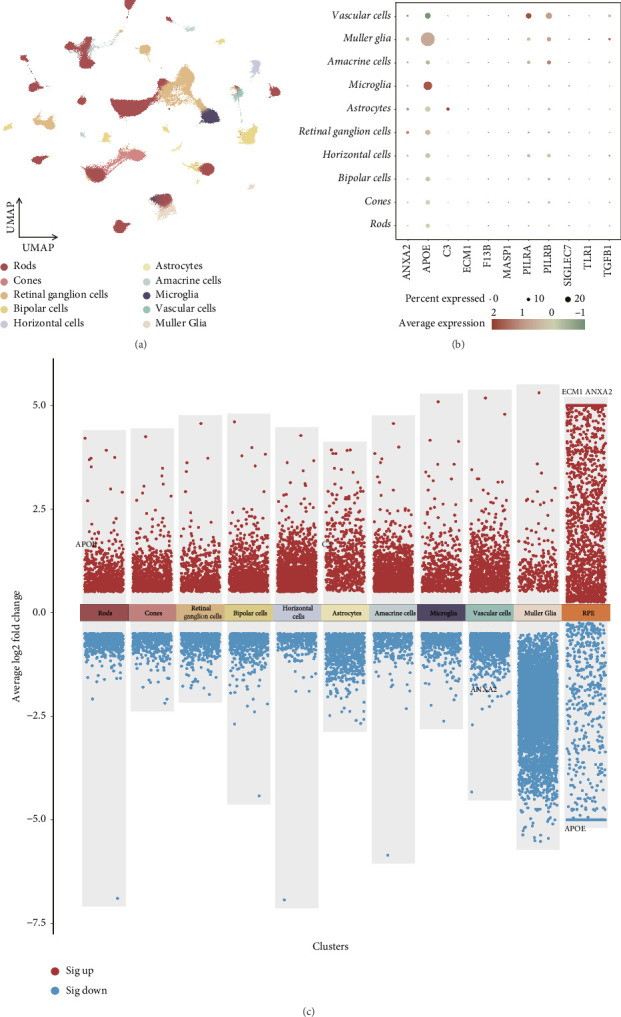
scRNA-seq delineates cell-specific expression of 12 plasma proteins in dry AMD retinal tissue. (a) Cell type distribution in retinal tissue, including 10 cell types. (b) Expression levels of 12 plasma proteins across different retinal cell types in dry AMD tissue. (c) The comparative gene expression analysis of 12 candidate proteins across a spectrum of 11 retinal cell types in both healthy and dry AMD tissues. Label genes: Log2FC > 0.5 and FDR < 0.05.

To investigate the retinal cell–type specificity of the 12 plasma proteins implicated in AMD, we performed a single-cell expression analysis utilizing scRNA-seq data using GSE221042 dataset. We identified various neuronal cell types, including retinal ganglion cells, horizontal cells, bipolar cells, rod photoreceptors, cone photoreceptors, and amacrine cells, as well as rare non-neuronal cell types such as microglia, astrocytes, Müller glia, and vascular cells (Figure 3(a)). Among the 12 causal proteins associated with dry AMD, genes corresponding to 11 retinal cell–specific genes were identified, CFHR2 being the only gene that was not detected (Figure 3(b)). Notably, APOE exhibited higher expression in Müller glia cells (Log_2_FC = 1.04, FDR < 0.05) and microglia (Log_2_FC = 1.64, FDR < 0.05). ANXA2 showed elevated expression in retinal ganglion cells (Log_2_FC = 0.55, FDR < 0.05), C3 in astrocytes (Log_2_FC = 3.03, FDR < 0.05), and PILRB in amacrine cells (Log_2_FC = 0.71, FDR < 0.05).

Given the pivotal role of RPE cells in AMD pathogenesis, we incorporated an additional scRNA-seq dataset for cross-validation (see Methods). This analysis identified four genes with significant cell type–specific enrichment in dry AMD tissues compared with healthy controls, each showing an average |Log_2_FC| > 0.5 and *p* < 0.05 (Figure 3(c)). Notably, APOE was enriched in rods and RPE cells, with adjusted *p* values also < 0.05. ANXA2 was upregulated in RPE cells but downregulated in vascular cells, ECM1 was upregulated in RPE cells, and C3 was upregulated in astrocytes. Overall, our scRNA-seq results revealed that APOE (in rods), ANXA2 (in vascular cells), C3, and ECM1 displayed expression patterns that were consistent with those identified through our MR analysis.

### 3.4. Assessing the Druggability Potential of 12 Candidate Drug Targets

**Figure 4:**
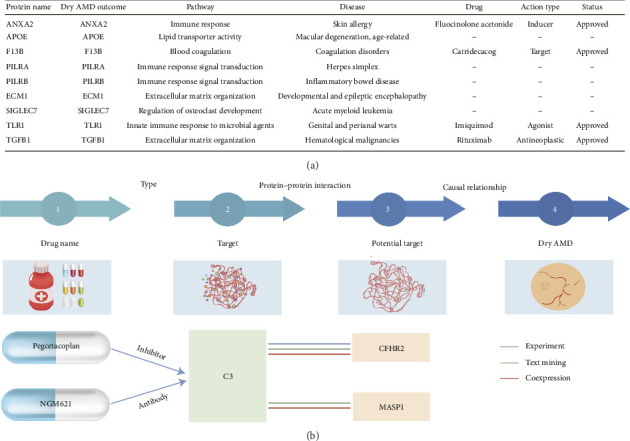
Classification and druggability evaluation of 12 dry AMD candidate drug targets. (a) Information on potential candidate targets for drug. (b) Interaction between dry AMD-associated proteins and identified dry AMD drugs.

**Figure 5:**
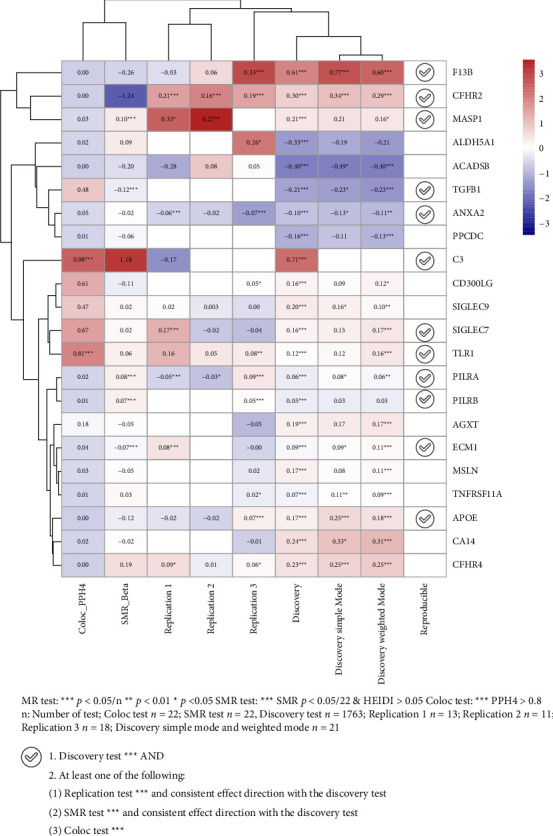
Sensitivity analysis validated the potential causal relationship between 12 proteins and dry AMD risk. Estimates from the meta-analysis of the discovery dataset and replication datasets for 12 proteome-wide identified proteins. Heatmap displays MR results only for proteins with qualified instruments (*F* > 10). Blank cells indicate proteins where no instruments met this threshold. Discovery dataset: UKB-PPP and FinnGen R11. Replication 1 dataset: deCODE and FinnGen R11; Replication 2 dataset: deCODE pQTL and Million Veteran Program (MVP) cohort; Replication 3 dataset: UKB-ppp pQTL and MVP cohort.

To facilitate the translation of genetic associations into therapeutic opportunities, we conducted in silico screening of 12 dry AMD-associated proteins (Figure 4(a)). Based on MR and pleiotropy analyses (Figure 5(a)), ten were classified as risk-associated and two as protective. These proteins span a range of biological pathways, including complement activation, coagulation, ECM organization, and immune regulation.

C3, a central effector of the complement cascade, is a well-established target in GA, with pegcetacoplan and NGM621 already approved or undergoing late-phase clinical evaluation [8, 55]. PPI further identified two upstream interactors, CFHR2 and MASP1, also involved in complement regulation, suggesting their potential as modulators within an expanded, targetable complement network [56] (Figure 4(b)). Beyond complement, several proteins operate within immune and coagulation systems. F13B, a coagulation factor targeted by catridecacog, may contribute to microvascular dysfunction in AMD, consistent with observed increases in retinal coagulation activity. TLR1, an innate immune receptor targeted by imiquimod, also represents a repurposing opportunity given the contribution of inflammation and microglial activation to AMD pathogenesis [57–59]. Among protective factors, ANXA2, a phospholipid-binding protein expressed in myeloid and Müller cells, was associated with a decreased risk of dry AMD [60]. Notably, fluocinolone acetonide, an approved corticosteroid for inflammatory skin disorders, acts as an inducer of ANXA2 expression, suggesting a potential regulatory axis involving ANXA2 in retinal homeostasis (Figure 4(a)). TGFB1 is notable for its roles in ECM remodeling and immunosuppression. Although primarily targeted by rituximab in hematologic diseases, its antifibrotic properties may hold relevance for GA management [61].

The remaining proteins, PILRA, PILRB, SIGLEC7, and ECM1, lack approved therapeutics but show enrichment in immune signaling and ECM pathways. Their expression in AMD-relevant retinal cell types supports further investigation as novel therapeutic candidates. Collectively, these ten risk-associated and two protective proteins span diverse biological pathways and druggability profiles, offering a structured roadmap for therapeutic inhibition or augmentation in dry AMD.

## 4. Discussion

In this proteome-wide MR and genetic pleiotropy analyses, we primarily uncovered 22 proteins with probable causal association with dry AMD. Through functional enrichment and PPI network analysis, we identified a network of 8 hub genes with pivotal interactive roles, indicating their significance in the dry pathogenesis of AMD. Following sensitivity analysis including the replication stage across three cohorts, colocalization, and SMR analysis, 12 drug target candidates were further validated. scRNA-seq was used to elucidate cell type–specific expression patterns in patients with dry AMD.

Our findings extend the well-established role of the complement cascade in AMD pathophysiology. Dysregulation of this innate immune pathway, especially overactivation of central components such as complement C3, has been extensively documented since the seminal discovery by Hageman et al., who linked CFH haplotypes to AMD susceptibility [62]. Through MR and Bayesian colocalization, we confirmed the causal effect of C3 and further identified upstream activators, including MASP1 and CFHR2 as risk factors. These findings support and extend the proteogenomic results reported by Emilsson et al., who identified complement-related proteins as convergent disease mediators across multiple conditions [63]. Notably, MASP1, a key initiator of the lectin pathway with high auto-activation potential, is already under investigation as a therapeutic target using SGMI-1 inhibitors [64]. While these insights underscore the therapeutic relevance of complement targeting, clinical experience with C3 and C5 inhibitors, namely, pegcetacoplan and avacincaptad pegol, reveals important safety concerns. In the phase III trials OAKS and DERBY, both agents were associated with an increased risk of endophthalmitis and systemic infections, likely due to broad complement suppression [65]. These risks are especially problematic given the role of the complement system in defending against encapsulated bacteria [66]. Moreover, there remains limited evidence of meaningful functional benefit, as visual acuity improvements were modest and long-term safety remains poorly characterized. These considerations reinforce the need for next-generation complement inhibitors with better safety profiles and mechanistic specificity.

Beyond complement, we identified several novel AMD-associated proteins with distinct functional profiles—such as F13B and TLR1—as novel risk candidates. F13B, a thrombin-activated transglutaminase involved in clot stabilization, may reflect a microvascular dysfunction component in AMD pathogenesis, consistent with histopathologic reports of choriocapillaris rarefaction in geographic atrophy [67]. TLR1, a microbial pattern recognition receptor, is targeted by imiquimod and underscores the possible role of innate immunity and retinal microglial activation [68, 69]. These risk-related proteins are attractive for inhibitory approaches and may support drug repurposing efforts from other inflammatory and coagulative disorders.

Among protective proteins, ANXA2 emerges as a compelling candidate. While previously linked to retinal angiogenesis and inflammation through the PI3K/AKT pathway, its protective association in dry AMD suggests a context-specific role [70–73]. In the non-neovascular setting, ANXA2 may exert anti-inflammatory or neuroprotective effects, potentially by preserving vascular stability or modulating subretinal immune responses. The observation that fluocinolone acetonide, a glucocorticoid with anti-inflammatory activity, acts as an inducer of ANXA2 expression further supports its putative role in inflammation resolution and retinal homeostasis. TGFB1 emerged as a compelling target. Known for its roles in ECM homeostasis and immunosuppression, TGFB1 has been implicated in retinal fibrosis and tissue remodeling [74]. Its protective association suggests a potential role in maintaining retinal immune quiescence or promoting early reparative remodeling. However, excessive TGFB1 signaling can also drive pathological fibrosis, highlighting the need for context-specific modulation rather than systemic augmentation.

The comprehensive nature of this study also addresses prior gaps in the AMD proteogenomic literature. Compared with earlier efforts such as Emilsson et al. [63], who established large-scale serum protein-disease associations, our approach directly connects genetically anchored plasma protein levels with AMD risk using two-sample MR and replication. Similarly, prior metabolomic studies have implicated HDL-related pathways in AMD [22], which our MR analysis also supported via APOE, though colocalization signals were modest. Jingzhi et al. [23] additionally demonstrated HDL-C and CRP as risk factors for AMD using NHANES-based MR. Our findings refine these metabolic associations by identifying upstream regulatory proteins potentially amenable to therapeutic manipulation. While our colocalization analysis identified strong evidence of shared causal variants for proteins such as C3 and TLR1 (PPH4 > 0.8), most other proteins did not exceed this threshold. However, this does not undermine the validity of the MR findings, as we used stringent LD clumping (*r*^2^ < 0.001, 10,000 kb) to ensure instrument independence and minimize confounding from correlated variants. Rather than serving as validation criteria, the colocalization results in our study are interpreted as supportive evidence for potential biological pleiotropy, where the same genetic variant may influence both protein levels and disease risk. In this context, the replication MR tests remain our primary evidence for robustness.

Despite these advances, several limitations should be acknowledged. We now summarize these in a dedicated limitations table (Table 2). Importantly, while MR strengthens causal inference by leveraging genetic instruments, it cannot definitively establish causality. Our use of cis-pQTLs which enhances interpretability and reduces the risk of horizontal pleiotropy, emerging evidence highlights the importance of trans-pQTLs in uncovering protein–disease relationships [75, 76]. Future studies incorporating trans-pQTLs may therefore uncover additional relevant proteins. In addition to the UKB-PPP and deCODE datasets used in our primary analysis, other independent pQTL resources, including SomaScan-based studies (INTERVAL, ARIC) and Olink consortia (SCALLOP, HUNT), could serve as valuable validation datasets to assess potential platform-specific measurement biases [77]. Furthermore, colocalization frameworks such as susieR or fine-mapping tools may help refine causal inference in future work [78]. Notably, our single-cell RNA-seq analyses, though valuable for exploring cell-type specificity, were limited by moderate donor numbers and batch-correction artifacts. Conclusions drawn from scRNA-seq should thus be viewed as exploratory and warrant validation in larger datasets. Finally, in the context of therapeutic translation, several associated proteins currently lack known drug modulators or detailed mechanistic characterization. Further functional studies are needed to evaluate their druggability and biological relevance in the pathogenesis of AMD.

Clinically, our results provide a rational framework for prioritizing therapeutic strategies. Among the 12 high-confidence proteins, six are already targeted by approved drugs, including C3, F13B, TLR1, CFHR2, MASP1, and ANXA2, offering immediate opportunities for drug repurposing. Importantly, we propose a stratified therapeutic model in which risk-associated proteins such as C3, F13B, and TLR1 may be inhibited, whereas protective proteins such as ANXA2 and TGFB1 may benefit from augmentation or mimetic approaches.

## 5. Conclusions

Extensive MR and sensitivity analyses across multiple cohorts identified 12 putative causal plasma proteins associated with dry AMD. The scRNA-seq elucidated the specific patterns of these proteins in patients with dry AMD. Furthermore, we classified these candidates to assess their druggability, interactions with existing drugs, and potential for polypharmacology.

## Figures and Tables

**Table 1:** Summary supportive evidence from Mendelian randomization (MR), colocalization, and SMR for 12 proteome-wide MR-identified proteins.

Protein	Protein full name	MR			Colocalization	SMR		
		OR (95% CI) discovery	*P*discovery	*P*replication	PPH4	Beta	*p*	*P*HEIDI
ANXA2	Annexin A2	0.90 (0.87–0.94)	2.74E − 06	8.39E − 07	4.77E − 02	−1.32E − 01	4.49E − 02	3.92E − 01
APOE	Apolipoprotein E	1.19 (1.13–1.26)	1.85E − 10	1.88E − 09	8.84E − 05	2.42E − 01	1.51E − 03	1.19E − 03
C3	Complement C3	2.04 (1.56–2.66)	1.87E − 07	9.10 − E1	9.83E − 01	7.12E − 01	8.79E − 07	1.74E − 03
CFHR2	Complement factor H related 2	1.36 (1.27–1.46)	4.74E − 20	9.58E − 13	2.45E − 20	2.83E − 01	1.09E − 03	3.07E − 23
ECM1	Extracellular matrix protein 1	1.09 (1.06–1.13)	9.37E − 08	1.01E − 05	3.63E − 02	7.01E − 02	2.23E − 01	6.44E − 01
F13B	Coagulation factor XIII B chain	1.84 (1.61–2.10)	3.71E − 18	2.80E − 06	5.97E − 95	5.92E − 02	3.29E − 01	1.37E − 87
MASP1	MBL-associated serine protease 1	1.23 (1.12–1.36)	1.38E − 05	4.80E − 03	3.45E − 02	1.08E − 01	2.20E − 01	4.13E − 01
PILRA	Paired immunoglobin like type 2 receptor alpha	1.06 (1.03–1.08)	8.55E − 07	6.90E − 13	2.27E − 02	1.30E − 02	8.23E − 01	6.80E − 02
PILRB	Paired immunoglobin like type 2 receptor beta	1.05 (1.03–1.08)	3.81E − 06	9.49E − 06	9.50E − 03	−1.88E − 02	7.38E − 01	1.71E − 01
SIGLEC7	Sialic acid binding Ig like lectin 7	1.17 (1.09–1.27)	5.93E − 06	8.32E − 04	6.68E − 01	2.81E − 01	1.08E − 03	7.08E − 01
TLR1	Toll like receptor 1	1.13 (1.06–1.22)	2.47E − 05	5.59E − 03	8.07E − 01	2.01E − 01	3.09E − 04	2.22E − 01
TGFB1	Transforming growth factor beta 1	0.81 (0.73–0.90)	1.07E − 05	NA	4.85E − 01	−2.26E − 01	2.06E − 03	1.59E − 01

**Table 2:** Limitations and implications of MR, colocalization, and SMR analyses in proteome-wide studies.

Category	Limitation	Implication
Causal inference with MR	MR strengthens causal inference but cannot confirm causality without experimental or clinical validation	Findings should be viewed as hypothesis-generating and require downstream validation
Instrumental variables (IVs)	Reliance on cis-pQTLs reduces horizontal pleiotropy but may miss trans-pQTLs with biological relevance	May limit the scope of protein coverage and underestimate true associations
Pleiotropy	Horizontal pleiotropy may still bias results, particularly for proteins influenced by immune or metabolic pathways	Conservative LD clumping and MR sensitivity analyses help mitigate but cannot eliminate this concern
Platform-specific bias	Differences in measurement platforms (e.g., Olink vs. SomaScan) may result in inconsistent quantification of protein levels	Results should be validated using orthogonal proteomic datasets (e.g., SCALLOP, ARIC)
Colocalization resolution	Coloc assumes a single causal variant per locus; this may be suboptimal for regions with multiple signals	Newer approaches such as SuSiE-R or FINEMAP may improve resolution
Replication strategy	MR replication was conducted using the MVP dry AMD GWAS. Additional replication in non-European or multi-ethnic populations would enhance generalizability	Results may not be directly generalizable to other ancestries
scRNA-seq sample size	The GSE221042 dataset includes a limited number of donors and may not capture full inter-individual variation	Cell-type–specific expression estimates may be subject to dropout, batch effects, and noise
scRNA-seq QC and assumptions	Quality control procedures for scRNA-seq were limited; doublet removal, donor-level stratification, and advanced batch correction were not performed. TPM normalization may also be suboptimal for UMI data	Interpretation of expression profiles should be considered exploratory and may contain residual technical noise
Drug target information	Some associated proteins lack known drug modulators or mechanistic studies	Their therapeutic potential requires further functional characterization and target prioritization

## Data Availability

The data that support the findings of this study are available in the supporting Information of this article.

## Supporting Information

Supporting Information 1 Supporting Table 1. Summary of pleiotropy analyses, heterogeneity tests, and causal results in the UKB-ppp pQTL and FinnGEN cohort.

Supporting Information 2 Supporting Table 2. Summary of 22 putative causal proteins identified by proteome-wide Mendelian randomization analysis in Discovery cohort.

Supporting Information 3 Supporting Table 3. Summary of pleiotropy analyses, heterogeneity tests, and causal results in the deCODE pQTL and FinnGEN cohort.

Supporting Information 4 Supporting Table 4. Summary of pleiotropy analyses, heterogeneity tests, and causal results in the deCODE pQTL and MVP cohort.

Supporting Information 5 Supporting Table 5. Summary of pleiotropy analyses, heterogeneity tests, and causal results in the UKB-ppp pQTL and MVP cohort.

Supporting Information 6 Supporting Table 6. Confirmed causality between 11 proteins and dry AMD in both discovery and replication cohorts.

Supporting Information 7 Supporting Table 7. Minimum and mean F-statistics for all prioritized proteins in both discovery and replication tests.

Supporting Information 8 Supporting Table 8. MR-PRESSO results in the Discovery and Replication phases.

Supporting Information 9 Supporting Table 9. Results of colocalization for putative causal plasma proteins identified in the Discovery phase.

Supporting Information 10 Supporting Table 10. Results of SMR for putative causal plasma proteins identified in the Discovery phase.

Supporting Information 11 Supporting Table 11. STROBE-MR-checklist.
